# Haplotype information of large neuromuscular disease genes provided by linked-read sequencing has a potential to increase diagnostic yield

**DOI:** 10.1038/s41598-024-54866-4

**Published:** 2024-02-21

**Authors:** Johanna Lehtonen, Anna-Maija Sulonen, Henrikki Almusa, Vilma-Lotta Lehtokari, Mridul Johari, Aino Palva, Anna H. Hakonen, Kirmo Wartiovaara, Anna-Elina Lehesjoki, Bjarne Udd, Carina Wallgren-Pettersson, Katarina Pelin, Marco Savarese, Janna Saarela

**Affiliations:** 1 Centre for Molecular Medicine Norway (NCMM), University of Oslo, Oslo, Norway; 2 Institute for Molecular Medicine Finland (FIMM), HiLIFE, University of Helsinki, Helsinki, Finland; 3 Folkhälsan Research Center, Folkhälsan Institute of Genetics, Helsinki, Finland; 4 Medicum, Faculty of Medicine, University of Helsinki, Helsinki, Finland; 5 Harry Perkins Institute of Medical Research, Centre for Medical Research, University of Western Australia, Nedlands, WA Australia; 6 Clinical Genetics, Helsinki University Hospital, Helsinki, Finland; 7 Molecular and Integrative Biosciences Research Programme, Faculty of Biological and Environmental Sciences, University of Helsinki, Helsinki, Finland; 8 Department of Medical Genetics, Oslo University Hospital, Oslo, Norway

**Keywords:** Genetics, Diseases, Medical research

## Abstract

Rare or novel missense variants in large genes such as *TTN* and *NEB* are frequent in the general population, which hampers the interpretation of putative disease-causing biallelic variants in patients with sporadic neuromuscular disorders. Often, when the first initial genetic analysis is performed, the reconstructed haplotype, i.e. phasing information of the variants is missing. Segregation analysis increases the diagnostic turnaround time and is not always possible if samples from family members are lacking. To overcome this difficulty, we investigated how well the linked-read technology succeeded to phase variants in these large genes, and whether it improved the identification of structural variants. Linked-read sequencing data of nemaline myopathy, distal myopathy, and proximal myopathy patients were analyzed for phasing, single nucleotide variants, and structural variants. Variant phasing was successful in the large muscle genes studied. The longest continuous phase blocks were gained using high-quality DNA samples with long DNA fragments. Homozygosity increased the number of phase blocks, especially in exome sequencing samples lacking intronic variation. In our cohort, linked-read sequencing added more information about the structural variation but did not lead to a molecular genetic diagnosis. The linked-read technology can support the clinical diagnosis of neuromuscular and other genetic disorders.

## Introduction

Molecular genetic analysis of large genes is demanding although high-throughput sequencing (HTS) has facilitated the task immensely. Rare or novel missense variants in large muscle genes such as *TTN* and *NEB* are common in the general population. The reconstruction of haplotypes (phasing) shows whether the variants are either *in cis* (on the same allele) or *in trans* (on two different alleles). Lack of phasing information makes variant interpretation difficult especially in sporadic adult-onset cases or if a patient has a de novo variant. Most diagnostic exome or genome sequencing analyses are not run as trios and phasing information is thus missing. If parental samples are not available, determining phasing information with traditional methods such as cloning is time-consuming and laborious. It should not be assumed that two truncating or likely pathogenic variants are always inherited from different parents. This might lead to mis-interpretation of variants or mis-diagnosis of patients^1^.

Short-read HTS collapses a diploid genome into a single sequence, but the long-read technology provides phase information of long continuous DNA fragments and resolves the haplotypes of patients. Haplotype information aids in the interpretation of putative disease-causing biallelic variants and thus supports genetic diagnostics. Long-read technologies, e.g. Pacific Biosciences’ (PacBio) single-molecule real-time (SMRT) sequencing^2^, Oxford Nanopore Technologies’ (ONT) nanopore sequencing^3^, and linked-read sequencing technology^4^ are actively used in research, but their clinical use has been more limited. Contrary to single-molecule sequencing technologies (PacBio and ONT), in linked-read technology, long-range information is achieved synthetically using bioinformatics to link barcoded short-read sequences derived from one DNA molecule to another in the data-analysis step, allowing multiple variants in the same gene to be phased^4^. Short-read-based linked-read sequencing offers a cost-effective way to achieve phasing information from a single DNA sample without the need for trio sequencing^5^, and its phasing ability has recently been successfully used also in diagnostics^6^.

Some of the genes causing neuromuscular disorders (NMDs), e.g. *TTN* and *NEB*, are known to have difficult-to-sequence repetitive genomic regions and they are partly “NGS dead zone” genes^7^. Thus, linked-read sequencing has the potential to improve the sequencing quality of such genes^4^. In addition to copy number variants (CNVs), long-range information of linked-read sequencing enables the identification of structural variants (SVs), i.e. inversions, based on the data. Linked-read sequencing has been shown to benefit the identification^8^ and phasing^9^ of SVs, and genotyping of short tandem repeat expansions^10^. It also enables de novo assembly of the human genome^11^.

Here, we report how linked-read sequencing succeeded to phase variants in a real-world setting, in ten autosomal genes, with the longest coding regions in the human genome associated with skeletal muscle disorders. In addition, we report how linked-read data performs in SV analysis. We also discuss the technical limitations of the method, such as the quality standards of DNA samples, which can present a problem in clinical diagnostics and need to be considered to obtain the best possible results.

## Results

### Phasing analyses

**Figure 1:**
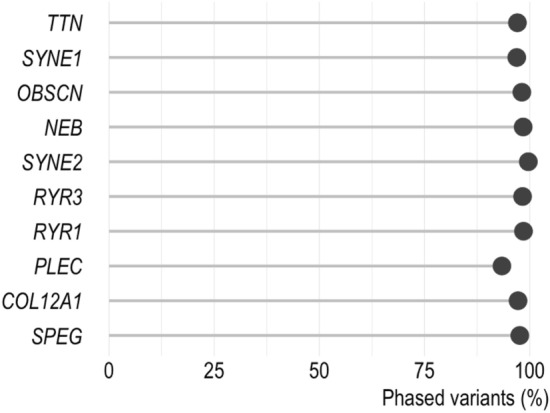
Variant phasing in large skeletal muscle genes. The figure illustrates that variant phasing is successful using the linked-read sequencing technique. On average, 97.5% of the exonic and splicing variants were phased in the ten largest skeletal muscle genes.

**Figure 2:**
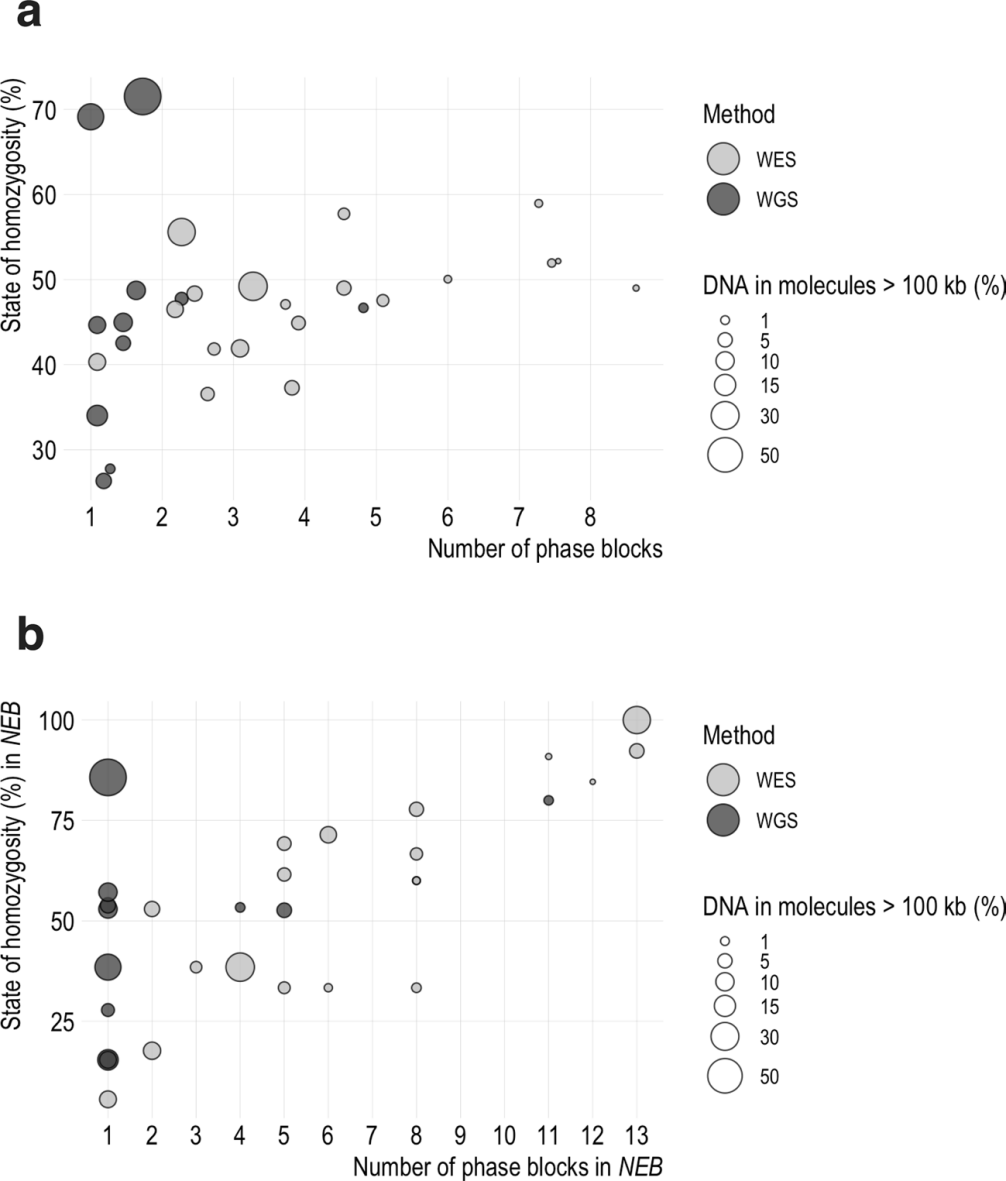
Fragmented or low-quality DNA, a high degree of homozygosity, and a selected sequencing method can increase a phase block number. (**a**) The plot shows the average state of homozygosity and the average number of phase blocks in the ten largest skeletal muscle genes for each sample. The size of the dot indicates the percent of long (> 100 kb) DNA molecules in the sample based on LongRanger calculation and the color of the dot expresses the used sequencing method. Our data show that fragmented or low-quality DNA or other sample processing related factors increased phase block number more than a high degree of homozygosity. (**b**) Despite long DNA fragments, *NEB* was divided into 13 phase blocks in two samples. Homozygosity can increase the number of phase blocks, especially in whole exome sequenced samples where intronic variation is lacking. Overall, whole genome sequenced (WGS) samples have fewer phase blocks than whole exome sequenced (WES) samples.

Linked-read sequencing was performed for 13 NMD patients and 17 control samples. An examination of exonic and splicing variants of the 30 samples shows that variant phasing is successful in large skeletal muscle genes: on average 97.5% of variants in the ten genes are phased (Fig. 1). The phasing was most successful in *SYNE2,* as 99.7% of its variants were phased, and most poor in *PLEC*, but still, on average 93.4% of its variants were phased successfully. Albeit the phasing of individual variants succeeded, haplotypes can only be reconstructed for multiple variants if they are in the same phase block. For the interpretation of variants, the most optimal is to have an entire gene of interest in the same phase block so that the haplotypes of both alleles are reconstructed for the entire gene. In variant phasing, the smaller number of phase blocks the better, since this indicates that long stretches of DNA are phased successfully. The calculated average state of homozygosity and the average phase block number of the ten largest skeletal muscle genes are plotted against each other in Fig. 2a. The dot size illustrates the algorithm-calculated DNA fragment length. The samples with the lowest percentage of long-stretched gDNA (i.e., the smallest dots in Fig. 2a) show the greatest number of phase blocks. The results imply that highly fragmented or otherwise lower quality DNA increases phase block number more than a high state of homozygosity. Figure 2a also shows that genome sequenced samples appear to perform better by having fewer and longer phase blocks compared with exome sequenced samples, even when the algorithm-calculated percentage of long DNA molecules is low. The genome sequenced sample with the largest average phase block number (4.8) has the lowest calculated percentage of long DNA fragments compared with other genome sequenced samples, but interestingly, it is not the one sample lacking size selection. Only 1.3% of DNA molecules were calculated to be more than 100 kb long in this whole genome sequencing (WGS) sample. The next smallest value (1.4%) in the WGS sample did not differ significantly from 1.3%, but what distinguishes the WGS sample with the largest average phase block number from the other WGS samples is not only the lack of ultra-long molecules but—based on the algorithm—the DNA was overall more fragmented: in that sample, only 25.4% of DNA molecules were over 20 kb while in the other WGS samples, the same value was in the range 61–98.1%. Investigation of the phase block number in individual genes showed that a high state of homozygosity increased the number of phase blocks, especially in exome sequenced samples lacking intronic variation. If the sample has no heterozygous variation, the phasing cannot be completed. In this cohort, the result was most evident in *NEB* (Fig. 2b). There were two whole exome sequencing (WES) samples with 13 phase blocks in *NEB* even though the DNA in those samples was not highly fragmented or low quality based on the algorithm. This was also observed in *OBSCN* (one WGS and one WES sample) and *RYR1* (one WES sample) (Supplementary Fig. S1c and f. online, respectively). Again, overall WGS samples performed better than WES samples and had fewer phase blocks.

### DNA integrity analysis

As DNA integrity or quality had a clear effect on the number of phase blocks after linked read sequencing, we evaluated the correlation between the measured DNA quality score and the LongRanger algorithm calculated percentage of long DNA molecules (> 20 kb and > 100 kb) and N50 phase block length. Supplementary Table 1 lists a Genomic Quality Score (GQS), the percentage of long DNA molecules, and the N50 phase block length for all the samples. There was no correlation between the GQS and the calculated percentage of long DNA molecules (> 20 kb or > 100 kb) or with N50 phase block length suggesting that DNA integrity does not alone define the phase block length (Supplementary Fig. S2a–c). The selected method had a greater impact on the N50 phase block length than the GQS. Genome sequenced samples had larger N50 phase blocks than the exome sequenced samples.

### Identification of pathogenic variants

**Figure 3:**
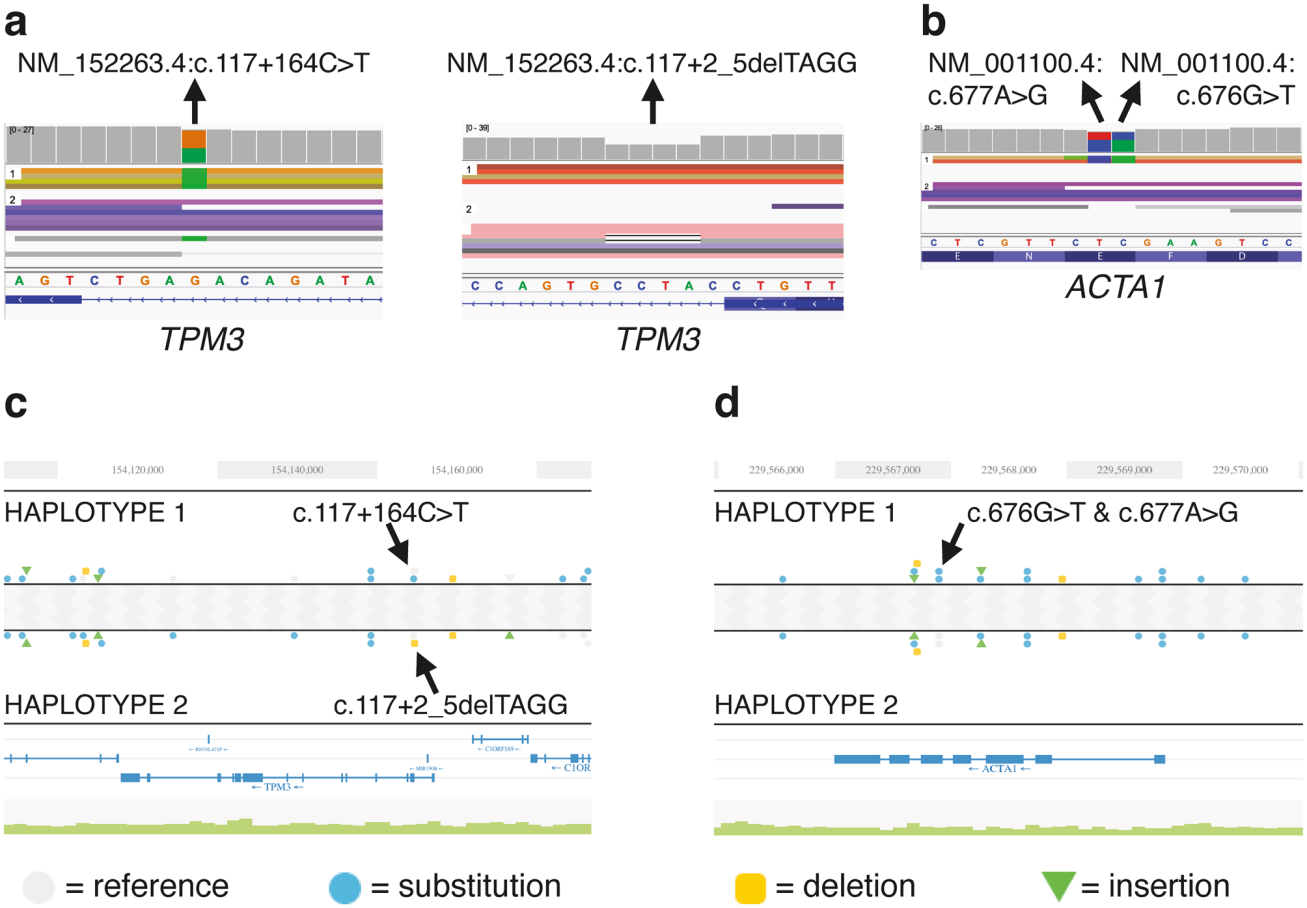
The linked-read IGV visualization shows that (**a**) intronic *TPM3* variants of patient 1 are biallelic, and that (**b**) two *ACTA1* variants of patient 2 are in the same allele. The haplotype view of Loupe software (10 × Genomics) also confirms that (**c**) two *TPM3* variants are biallelic, and that (**d**) both identified *ACTA1* variants are in the same allele.

Table 1 lists phenotype descriptions of all the patients included in the study. More detailed phenotype descriptions of unsolved patient cases are provided in “Supplementary Information” online. Two patients with a diagnosis of nemaline myopathy were included in the study. Patient 1 had undergone several molecular genetic analyses before linked-read sequencing: Sanger sequencing of nemaline myopathy genes (including *TPM3*), short-read WES, and targeted array-based Comparative Genomic Hybridization (array-CGH)^12^. Linked-read sequencing identified biallelic intronic variants in *TPM3*, NM_152263.4:c.117 + 2_5delTAGG and NM_152263.4:c.117 + 164C > T, which have been previously detected using Sanger sequencing (Fig. 3a and c). The significance of the intronic variant NM_152263.4:c.117 + 164C > T remained uncertain until RNA sequencing revealed that both variants affect the splicing of *TPM3* intron 1a^13^. Patient 2 had received no molecular genetic verification of his clinical and histological diagnosis of nemaline myopathy. Previously, an array-CGH was conducted for the sample^14^. Using linked-read sequencing, an *ACTA1* variant NM_001100.4:c.676_677delGAinsTG was identified in the patient sample (Fig. 3b and d). A change of two nucleotides in the same allele and the same codon resulted in a novel missense variant NP_001091.1:p.(Glu226Trp). The identified p.(Glu226Trp) is presumably a de novo pathogenic variant in the patient (no sample of the patient’s healthy father was available, and the variant was not identified in the healthy mother). Previously, p.(Glu226Gln) and p.(Glu226Gly) have been reported as pathogenic variants causing dominantly inherited nemaline myopathy^15^. All the variants were verified by Sanger sequencing. Patient 4 with distal myopathy was included in this study as a control. Patient 4 had a previously identified pathogenic variant NM_001103:c.2567del:p.(Pro856Argfs*45) in *ACTN2*^16^. This variant was also identified using linked-read sequencing hence the usage of this method would have offered a molecular genetic diagnosis for the patient.

**Table 1:** A summary of patients included in the study.

Sample	Sex	Onset	Occurrence	Parental sample(s) available	Muscle weakness
1	M	Congenital	Sporadic	Both	Nemaline myopathy, upper limbs weaker than lower^13^
2	M	Congenital	Sporadic	One	Nemaline myopathy, lower limbs weaker than upper**
3	F	Congenital	Sporadic	Both	Global muscle weakness, cognitive difficulties**
4	F	Adult	Familial	None*	Distal myopathy with facial weakness. Slowly progressive^16^
5	M	School age	Familial	Both	Proximal and distal lower limbs. Progressive^17^
6	M	Adult	Familial	Both	Distal myopathy, lower legs weaker than hands. Slowly progressive^18^
7	M	Adult	Sporadic	None	Distal myopathy**
8	M	Adult	Unclear	None	Distal myopathy**
9	F	Adult	Sporadic	None	Proximal myopathy**
10	F	Late adult	Sporadic	None	Distal myopathy, lower legs weaker than hands**
11	M	Late adult	Sporadic	None	Distal myopathy, lower legs weaker than hands**
12	M	Adult	Sporadic	None	Distal myopathy, lower legs weaker than hands**
13	M	Late adult	Sporadic	None	Distal myopathy, lower legs weaker than hands**

*A DNA sample of the patient’s daughter was available.

**More information is included in the “Supplementary Information”.

### Structural variant analysis

Supplementary Table S2 online summarizes the structural variant results. The short-read-based CNV callers (DECoN and copyCat) called 307 unfiltered CNVs in the NMD genes (a virtual gene panel of 343 genes; Supplementary Table S3 online) in ten WES samples. In comparison, the LongRanger pipeline called considerably fewer (n = 29) SVs. Moreover, the median length of the called SVs varied between the short and long-read-based callers: 17 kb vs. 171 bp, respectively. Only one large SV call from the LongRanger pipeline, identified as two shorter calls in the short-read-based CNV pipeline, was identified by both methods when a reciprocal minimum 20% overlap was required (Supplementary Fig. S3a online). The number of called SVs was more even in WGS samples: copyCat called 77 CNVs and LongRanger 93 SVs, and the median lengths were 2 kb vs. 308 bp, respectively. Nonetheless, only six of the SVs were identified with both methods (Supplementary Fig. S3b online). Many of the SV calls from both pipelines were a common variation or likely false positive calls, based on the quality values and IGV visualization. After filtering, none of the other identified SVs strictly matched the phenotype of the patient, nor the validation of the SV was successful (Supplementary tables S4–S7 online). Thus, no novel disease-causing SVs were identified in this study. However, the study included two NMD patient samples with previously identified deletions as controls. The first sample with the previously identified SV (patient 5) carried a heterozygous *TTN* deletion of exons 34–41^17^. It was not identified by the LongRanger pipeline but was identified by using our short-read-based CNV calling pipeline using linked-read WES data (Supplementary Table S4 online). The heterozygous *HNRNPA1* deletion of exon 10^18^ of the second sample with known SV (patient 6) was neither identified with our own CNV pipeline nor with the LongRanger pipeline from linked-read WES data. In Patient 1 the LongRanger pipeline called a putative heterozygous 48.5 kb long inversion chr11:1,915,271–1,961,063 (hg19 coordinates), including the entire *TNNT3* gene, which is associated with recessive nemaline myopathy (Supplementary Table S7/Fig. S4 online). However, this inversion was not considered to be pathogenic, at least in the heterozygous state, because there are several inversions reported in the Database of Genomic Variants (DGV) in the same region. Instead, the above-mentioned biallelic *TPM3* splicing variants were considered to be causative in this patient.

## Discussion

As an improvement to a short-read HTS, long-read sequencing enables the phasing of variants^4^. We estimate that phasing information is missing in up to 70–75% of NMD patients. Based on our analysis, the linked read sequencing was able to phase 97.5% of variants in the very large NMD genes. The length of the individual phase blocks varied between the samples, and was influenced by the sequencing method, level of homozygosity, and quality of the DNA sample. The availability of the phasing information at the time of clinical genetic analysis makes the diagnostics potentially faster and cheaper. If it is clear from the data that two heterozygous variants in a gene are on the same allele, they can be excluded as the cause of a recessive disease when interpreting putative biallelic disease-causing variants. Segregation analysis may be time-consuming, especially if DNA samples of the family members are not collected at the same time as the sample from the index patient, further increasing the diagnostic turnaround time. Furthermore, samples from family members are not always available as in many of the cases investigated in this study.

Adequacy of ultra-low input material (as little as 0.1 ng of gDNA) for library construction is a major advantage of linked-read sequencing^5^ compared with ONT and PacBio sequencing which currently require 500–1000 ng and 6000–10,000 ng of gDNA for human WGS library construction, respectively. However, if a recommended gDNA size selection is performed prior to library preparation for linked-read sequencing samples, the required gDNA start material ranges usually between 100 and 200 ng depending on the sample quality. In this study, there was a limited amount of DNA available from many patients and therefore, native long-read sequencing methods could not be utilized. The size selection step and a dilution of a sample to an ultra-low concentration prolong the sample preparation time for linked-read sequencing. In contrast, for ONT and PacBio sequencing gDNA is fragmented and a sample does not need an extensive dilution. Hands-on time in the sample preparation, quality control (QC), and library processing is the shortest for ONT sequencing and all these steps can be performed on the same day. For PacBio sequencing these steps typically take two working days, and for linked read sequencing three to four working days including the gDNA size selection and a sample dilution. On-instrument sequencing time for a human genome is two days using ONT and linked-read sequencing but PacBio sequencing takes one to six days depending on the used instrument. Today, sequencing costs with similar read depth are more expensive using PacBio than ONT or short-read sequencing. Importantly, as linked-read sequencing is based on short-reads, it can be incorporated into diagnostic use without the need in investing a second sequencing instrument or set up an independent data analysis pipeline, making it a cost-effective way to achieve phasing information^5^. However, as the advent of new versions of the currently available native long-read sequencing instruments is likely to reduce the amount of the input DNA required and the cost of sequencing of a human genome, it is presumable that native long-read sequencing methods will become a standard method in the diagnostics of NMD patients in the future.

Somatic mosaicism for variants, e.g. in *ACTA1*^19^ and *NEB*^20^, have been shown to cause NMDs. Mosaic variants can be detected by HTS if the somatic variant is present in the sequenced tissue, and if the sample is sequenced in a sufficient read depth. Today, identifying mosaic variants would be more feasible by linked-read sequencing than by ONT or PacBio sequencing because of the lower input material and because deep coverage sequencing is more cost-efficient by using short reads. Linked-read sequencing data can be analyzed without the additional long-range information as regular short-reads, which enables running it through pipelines targeted for somatic SNV or small indel detection. Also, LinkedSV, an algorithm developed for linked-read sequencing data, has successfully identified mosaic structural variants with low allele frequency^8^.

Our results showed that linked-read WGS performs better than linked-read WES, as the longer phase blocks are easier to achieve even with DNA samples with a limited calculated percentage of long DNA fragments. However, for the DNA samples with the lowest percentage of long molecules based on the LongRanger algorithm, the number of phase blocks increases despite the usage of linked-read WGS. The intronic regions included in genome sequencing data have more heterozygous variation, helping to form longer phase blocks. Still, based on our results, the most important factor ensuring long phase blocks is the long DNA fragments in samples.

Interestingly, genome quality score (GQS) used to evaluate the integrity of the DNA samples did not correlate with the LongRanger calculated percentage of > 20 kb or > 100 kb DNA molecules or with the global N50 phase block length. For example, the lowest-performing WGS sample had the second-highest GQS of all the samples. This suggests that other factors, such as DNA purity and the efficacy of the linked-read library preparation protocol, are also likely to affect the data quality. Additionally, only two of the measured GQSs were below 4 (range 0–5; 5 indicating the most intact DNA) thus GQS did not clearly differentiate samples from each other. Furthermore, as we applied size selection to the DNA samples after GQS measurement and DNA fragments lower than 40 kb were unselected from the library process, the size range of the DNA sample used for library preparation did not directly reflect the size range of the original DNA sample.

In our collaborative study, the data was collected from different cohorts, thus the DNA extractions were done in different laboratories at different times using standard methods. Ideally, DNA extraction should be done from fresh samples with a method specifically designed for long-fragment extraction. This allows extraction of high molecular weight genomic DNA (HMW gDNA) which is preferably used in long-read HTS. However, this is not always possible in clinical diagnostics as it was not in our study. Size selection of long fragments for gDNA is always recommended, but this applies especially to samples which cannot be re-collected and re-extracted and are extracted with standard methods.

Our results indicate that homozygosity increases the number of phase blocks even when the DNA in the samples is not fragmented. The result was most pronounced for the *NEB* gene, but our data showed the same trend for *OBSCN* and *RYR1*. Due to the small sample size, and the fact that we were not able to control for DNA quality, it is difficult to conclude how frequently homozygosity would cause a problem in variant interpretation under standardized circumstances. However, if the DNA sample meets the quality standards and the library processing is successful, a high state of homozygosity would likely not cause difficulty in the phasing of compound heterozygous variants, since rare disease-causing variants would probably allow the gene to be phased.

We identified a novel disease-causing variant p.(Glu226Trp) in *ACTA1* in one patient. This missense variant was caused by two different nucleotide changes next to each other and linked-read sequencing phased them in the same allele. Because the nucleotide changes were next to each other, also short-read HTS would have shown them to be inherited in the same allele. However, short-read HTS can only reveal phasing if the nucleotide changes are less than a read length from each other.

The LongRanger pipeline gives as output files a variant call format (VCF) file for single nucleotide variants (SNVs) containing additional phase information, and a VCF of SVs, which can be annotated. Yet, the sequencing data can also be analyzed without the additional long-range information. We compared SV analysis based on short reads with and without long-range information: only one WES and six WGS calls were identified by both methods. Also, the number and length of identified SVs differed between the methods. This was somewhat expected due to the differences in the SV calling algorithms. Of the two previously identified deletions; analysis of the short-read data without long-range information using three CNV callers identified one of the deletions, while the analysis based on LongRanger pipeline data identified neither of them. The heterozygous *TTN* deletion of exons 34–41 identified previously^17^ was only found using short-read-based CNV analysis of linked-read data of patient 5. The DNA of the sample was good quality based on the GQS but highly fragmented according to LongRanger: only 22.2% of DNA molecules were > 20 kb and 0.3% were > 100 kb, resulting in *TTN* phased in 17 blocks (Supplementary Table S1 online). Both methods missed the short heterozygous deletion of 160 bp including only one exon^18^ from linked-read WES data. The deletion was most probably missed due to poor sequencing coverage in the deletion region (on average 5 × for the region). Also, copyCat and CODEX2 CNV callers require more than one affected exon for a CNV call. The deletion was identified with a subsequent high-coverage short-read genome sequencing^18^. Different pipelines lead to varying SV call results; thus combining the results of multiple callers in the analysis is recommended. Several linked-read-specific SV calling pipelines such as LinkedSV^8^ have been developed in addition to LongRanger. Although linked-read sequencing has been previously reported to lead to the identification of SVs missed using short-read sequencing^8,21^, linked-read sequencing did not significantly improve the SV identification in our study nor in the studies done by others^21,22^. Nevertheless, due to the long phase block length with optimal DNA samples (typically 50–100 kb), linked-read sequencing has been suggested to have the potential to identify large SVs better than other long-read methods (read length typically 20–30 kb)^8^. SVs located in repetitive regions are also better found using linked-read than short-read sequencing^21^. However, it is difficult to draw firm conclusions, as the sample sizes in the studies were relatively small.

In conclusion, linked-read sequencing can phase most of the variants in the very large NMD genes successfully. This is likely to shorten the time used in making a molecular genetic diagnosis of patients by providing the phase information for the identified variants in a cost-effective way. This method has potential to offer a diagnosis for unresolved patient cases. However, although linked read sequencing is utilizing short-read sequencing instruments typically available in clinical laboratories, bringing it into use in diagnostics requires pipeline optimization and high-quality long-fragment DNA for the method to reach its maximum potential.

## Methods

### Ethical approvals and patient consent

The study was approved by the Ethics Review Board of the Helsinki University Hospital (number 195/13/03/00/11) and performed according to the Declaration of Helsinki. Written informed consent was collected from all the patients and/or their legal guardians. All study methods were performed in accordance with relevant guidelines and regulations.

### Patient selection

Ten patients with genetically undiagnosed myopathies were selected in this study based on previously performed genetic studies being inconclusive. Many of the samples were singleton and familial samples were not available for segregation analysis (Table 1), therefore, phasing information was expected to bring additional information to the analysis. Linked-read sequencing suited well as a selected method because only a limited amount of DNA was available from majority of the samples (”Supplementary Information”).

### Sample preparation, linked-read sequencing, and variant annotation

**Figure 4:**
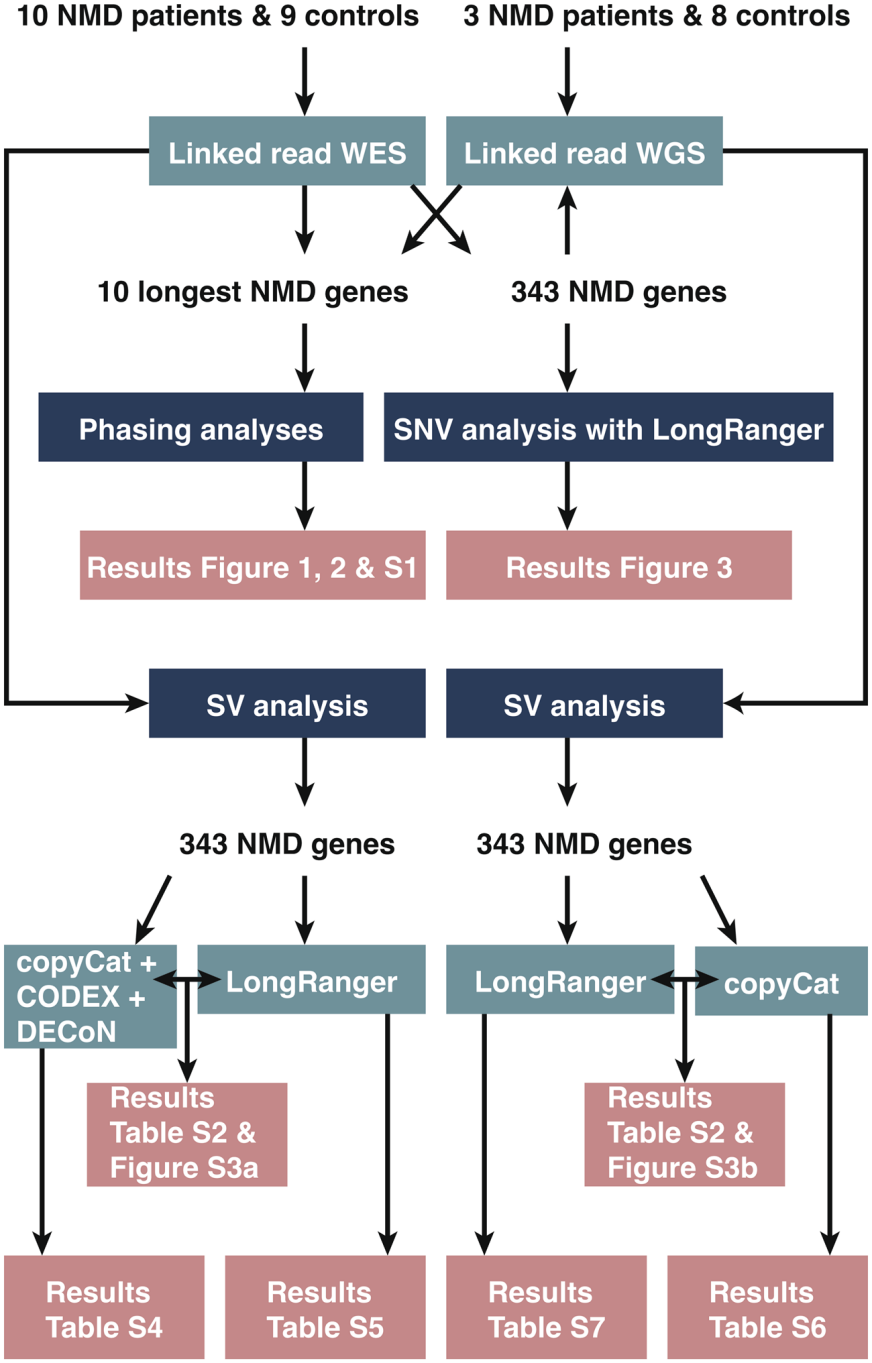
The flow chart of the analyses conducted in the study. Both linked read whole exome sequencing (WES) and whole genome sequencing (WGS) were used. The study was targeted at neuromuscular disorder (NMD genes). Single nucleotide variants (SNVs) and structural variants (SVs) were studied.

Figure 4 shows a flow chart of the study. Genomic DNA was extracted from EDTA-blood samples or myoblast cells using standard protocols. Linked-read sequencing was performed for 30 samples: WES for 19 samples and WGS for 11 samples. As a part of the sample QC, a GQS of six WGS samples and 19 WES samples were measured with a LabChip GX Touch machine (PerkinElmer, USA) and GQS was used to estimate DNA integrity. The GQS of the sample was plotted against the LongRanger calculated percentage of > 20 kb or > 100 kb DNA molecules or the global phase block N50 length (Supplementary Fig. S2a–c) using RStudio version 2023.09.0 based on R version 4.3.1. Next, a genomic DNA size selection was done for all except one genome control sample (sample 17) with the Sage Blue Pippin system (Sage Science, Beverly, MA, USA), using 40–80 kb as a selected range. Size-selected genomic DNA (gDNA) was used in library preparation. The linked-read WES libraries were processed according to Chromium Exome Demonstrated Protocol (Genome Reagent Kits v2 for Exome Assays CG000059 Rev C; 10 × Genomics, Pleasanton, CA, USA). In the protocol, steps 5.1–6.2 were replaced with NimbleGen SeqCap EZ Library SR User’s Guide v5.1 or v5.3 chapters 5–6 with the following modifications: 1000 ng per sample were pooled for each capture, blocking oligos were replaced with 10 μl of IDT xGen Universal Blockers—TS mix, and 25 µl of COT was used per sample. Linked-read WGS libraries were processed using Chromium Genome Reagent Kits v2 CG00043 Rev B (10 × Genomics, Pleasanton, CA, USA). Linked-read exome sequencing was performed on HiSeq 1500, HiSeq 2500, or NovaSeq 6000 platform (Illumina, San Diego, CA, USA) with paired-end 100 or 150 cycle runs (more information in Supplementary Table S1 online). The 11 linked-read genome samples were sequenced on the NovaSeq 6000 system (Illumina, San Diego, CA, USA) using S1 or S4 flow cell and XP workflow. The read length for a paired-end run was 150. The sequencing reads were analyzed using LongRanger v2.2.2 variant calling pipeline with default parameters and Genome Analysis Toolkit (GATK) for quality control, short-read alignment (GRCh37), and variant identification. The gDNA size selection, library preparation, sequencing, and variant calling were performed at the Technology Centre of the Institute for Molecular Medicine Finland (FIMM). After running the LongRanger pipeline, we added two additional filtering steps: all the variant calls, which were present in < 20% reads, and/or had coverage of < 7 reads, were filtered out. Variant annotation was performed using an in-house developed Annovar-based annotation pipeline^23^.

### Variant phasing

The analyses of the performance of linked-read technology included data from 19 WES samples and 11 WGS samples. Regardless of the allele frequency, all the exonic and splicing variants which passed the quality filtering steps were included in the phasing analyses. The analyses included variants in the ten autosomal genes with the longest coding sequences (Supplementary Table S8 online) associated with skeletal muscle disorders. The phasing analysis was focused on how linked read sequencing succeeded to phase variants and how DNA and data quality affected the number of phase blocks in the samples. The graphs were created with RStudio version 2022.07.0 based on R version 4.2.2.

### Variant identification

Three sequenced genomes and ten exomes were from NMD patients. The analysis was targeted at exonic and splicing variants. Synonymous variants, variants with minor allele frequency (MAF) > 0.01 in Genome Aggregation Database (gnomAD, USA), and variants occurring frequently in our in-house database were filtered out. The data was analyzed using a virtual gene panel for NMD including 343 genes (Supplementary Table S3 online). Phasing information and in silico prediction tools were utilized in variant prioritization. The putative disease-causing variants were verified by Sanger sequencing and their segregation was studied in family samples if they were available. Primers are available on request. Supplementary Table S9 (online) lists SNVs of NMD genes if there were multiple variants in the same gene, i.e. phasing was examined.

### Structural variant analysis

We performed CNV analysis using our in-house developed CNV analysis pipeline and LongRanger v2.2.2 SV calling output to compare the results. The sequenced samples included those of two patients with known deletions as controls. SVs of ten WES and three WGS NMD patients were analyzed. For both linked read WES and WGS data, CNVs were analyzed using 1) an in-house developed short-read-based CNV calling pipeline (copyCat, manuscript in preparation), and SVs with 2) linked-read-based LongRanger calling output. In addition to that, WES data was run through 3) Detection of Exon Copy Number (DECoN) version 1.0.2^24^, and 4) COpy number Detection by EXome sequencing (CODEX2) version 1.3.1^25^ pipelines for CNV identification. In copyCat, DECoN, and CODEX2 callers the patients’ sequence data is compared against a control set including data with similar read depths. CopyCat compares RPKM normalized^26^ read depths between a test sample and a median of the control samples on partitioned genomic regions. In WGS, the genome was partitioned into regions of 500 bp, and in WES the targeted exons were used as partitions. Partitions were then transformed to CNV calls by using a circular binary segmentation (CBS) from the R package DNAcopy version 1.50.1^27^. Output for the short-read-based WES analyses was CNVs identified with copyCat and/or DECoN, with possible overlap percent information for the CNVs identified with CODEX2. For the short-read-based WGS analysis, only copyCat was used for CNV identification, as DECoN and CODEX2 are only developed for WES-based data. All the identified SVs were then annotated for repetitive genome elements, overlapping genes, population frequencies, known disease associations, ACMG classification, and several in-house-built metrics using an in-house developed Annotation and Analysis of Structural Variants pipeline (AnAnaS, manuscript in preparation). Further SV analyses were targeted at the 343 NMD panel genes. For the Venn diagram comparison of the SVs identified with both short-read and linked-read-based methods, the overlapped region needed to be ≥ 20% of the SVs reciprocally. When we aimed to identify disease-causing SVs in the samples, the following filtering steps were used for all the SV data: calls overlapping telomeres or centromeres, calls overlapping ≥ 80% with repeat masked sequences, and calls that did not overlap with any of the 343 NMD panel genes were left out of the analysis. In addition, the following filtering criteria were used depending on the sequencing method and the pipeline used: (1) the short-read-based CNV analysis of WES data included only the calls overlapping exon regions, and the calls needed to be obtained with at least two different callers (copyCat, DECoN, or CODEX2) with an overlap of ≥ 20%. One of the samples (Patient 7) was left out of the analysis because of poor quality. All the 26 CNV calls fulfilling the criteria are listed in Supplementary Table S4 online. (2) The LongRanger v2.2.2 SV analysis of WES data included calls that either had a phase block number, i.e. phasing was successful, or had an overlap of ≥ 20% with a call from another caller (copyCat, DECoN, or CODEX2). All six SV calls fulfilling the criteria are listed in Supplementary Table S5 online. (3) The short-read-based CNV analysis of WGS data included CNVs if they were called < 4 times in the patient and control group and if the CNV size was ≥ 5 partitions (one partition = 500 bp). All the 38 CNV calls fulfilling the criteria are listed in Supplementary Table S6 online. (4) The LongRanger v2.2.2 SV analysis of WGS data included calls that either had a phase block number, i.e. phasing was successful, or had an overlap of ≥ 20% with a copyCat call. All the ten SV calls fulfilling the criteria are listed in Supplementary Table S7 online. After that, all the SV calls were manually prioritized based on the quality values, IGV visualization, population frequencies, phenotype information, ACMG classification, and the information on known disease variants in ClinVar and ClinGen overlapping an SV.

### Supplementary Information

Supplementary Information.

Supplementary Tables.

## Data Availability

The datasets generated during and/or analyzed during the current study are not publicly available because of privacy and legal restrictions. According to national and EU regulations health data, including genome sequences, can only be made publicly available if non-identifiable or specifically allowed by the research consent. The signed consents do not cover depositing data to a public data repository but cover access to the data for the research questions specified in the consents. Linked-read sequencing data of the neuromuscular disorder patients are available from the corresponding author (janna.saarela@ncmm.uio.no) for neuromuscular disorder-related research questions on request. Access to the data can be provided in a reasonable timeline in a controlled environment after signing the data access agreements of the institutes controlling the data.
