# Supplementary material for: Haplotype information of large neuromuscular disease genes provided by linked-read sequencing has a potential to increase diagnostic yield
Source: Sci Rep. 2024 Feb 21;14:4306. doi: 10.1038/s41598-024-54866-4 (PMC10881483; doi:10.1038/s41598-024-54866-4)
Supplement: Supplementary file 1 — Supplementary Information. [file 41598_2024_54866_MOESM1_ESM.pdf]

Supplementary information: Haplotype information of large neuromuscular disease genes provided by linked-read sequencing has a potential to increase diagnostic yield

Johanna Lehtonen[1,2,3,4], Anna-Maija Sulonen[2], Henrikki Almusa[2], Vilma-Lotta Lehtokari[3,4], Mridul Johari[3,4,5], Aino Palva[2], Anna H. Hakonen[6], Kirmo Wartiovaara[6], Anna-Elina Lehesjoki[3,4], Bjarne Udd[3,4], Carina Wallgren-Pettersson[3,4], Katarina Pelin[3,4,7], Marco Savarese[3,4], Janna Saarela[1,2,8]*

[1] Centre for Molecular Medicine Norway (NCMM), University of Oslo, Oslo, Norway

[2] Institute for Molecular Medicine Finland (FIMM), HiLIFE, University of Helsinki, Helsinki, Finland

[3] Folkhälsan Research Center, Folkhälsan Institute of Genetics, Helsinki, Finland

[4] Medicum, Faculty of Medicine, University of Helsinki, Helsinki, Finland

[5] Harry Perkins Institute of Medical Research, Centre for Medical Research, University of Western Australia, Nedlands WA, Australia

[6] Clinical Genetics, Helsinki University Hospital, Helsinki, Finland

[7] Molecular and Integrative Biosciences Research Programme, Faculty of Biological and Environmental Sciences, University of Helsinki, Helsinki, Finland

[8] Department of Medical Genetics, Oslo University Hospital, Oslo, Norway

Corresponding author:

j.s.saarela@ncmm.uio.no, janna.saarela@helsinki.fi

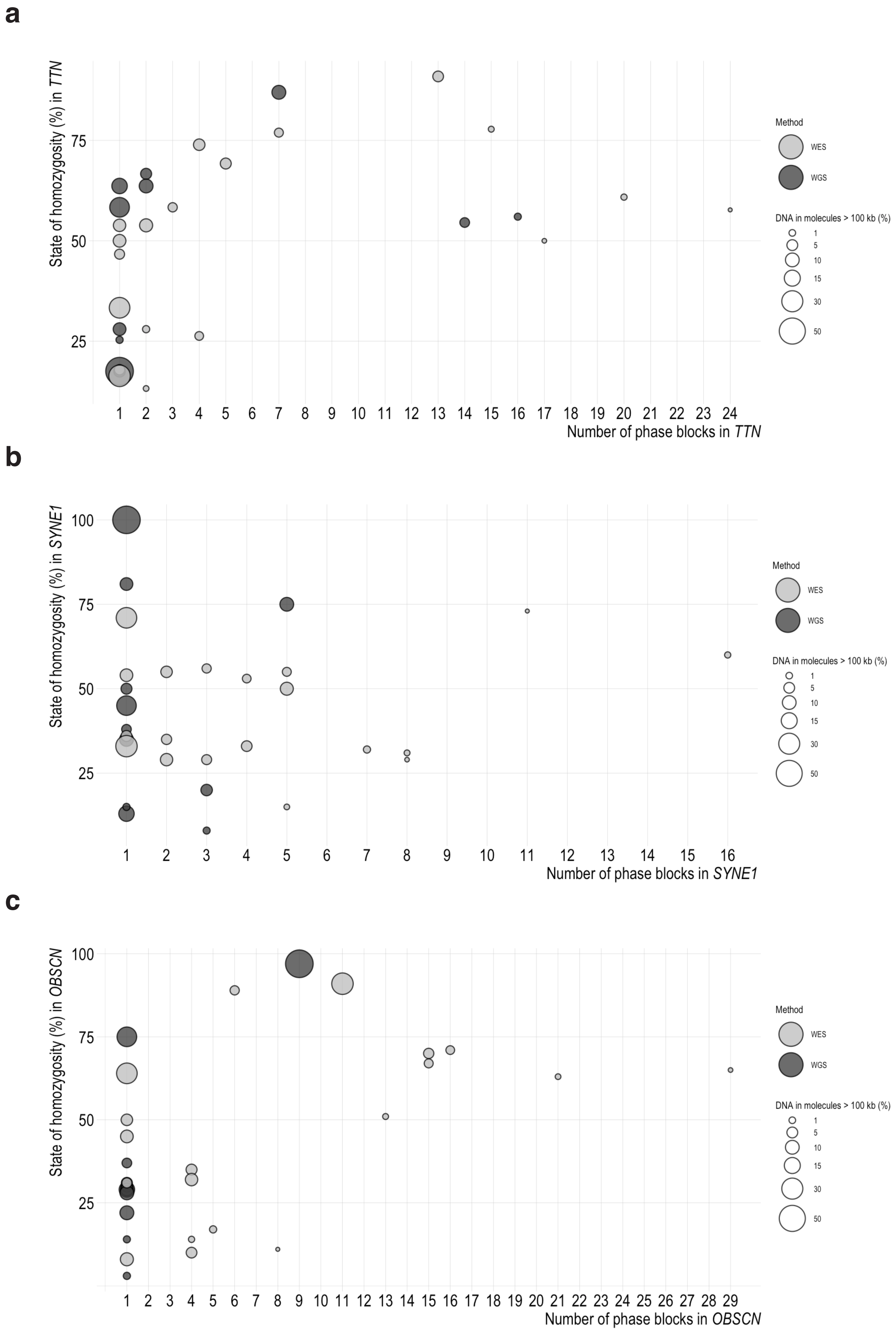
a
State of homozygosity (%) in TTN
Number of phase blocks in TTN
Method
WES
WGS
DNA in molecules > 100 kb (%)
1
5
10
15
30
50
b
State of homozygosity (%) in SYNE1
Number of phase blocks in SYNE1
Method
WES
WGS
DNA in molecules > 100 kb (%)
1
5
10
15
30
50
c
State of homozygosity (%) in OBSCN
Number of phase blocks in OBSCN
Method
WES
WGS
DNA in molecules > 100 kb (%)
1
5
10
15
30
50

d

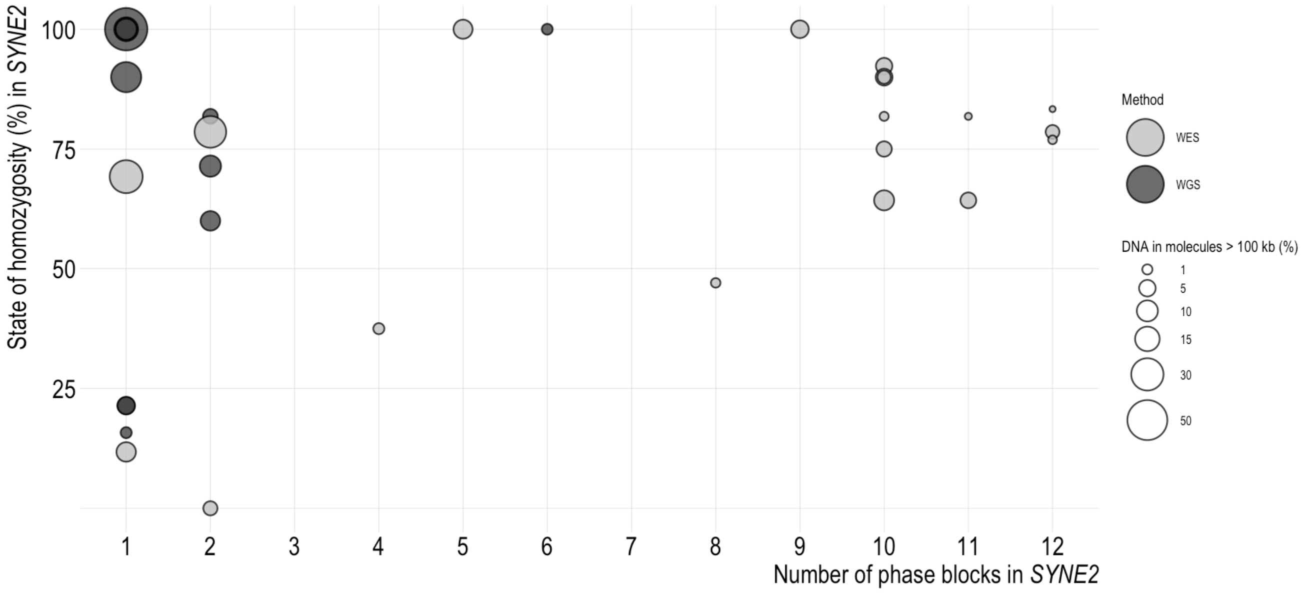
State of homozygosity (%) in SYNE2
100
75
50
25
1
2
3
4
5
6
7
8
9
10
11
12
Number of phase blocks in SYNE2
Method
WES
WGS
DNA in molecules > 100 kb (%)
1
5
10
15
30
50

e

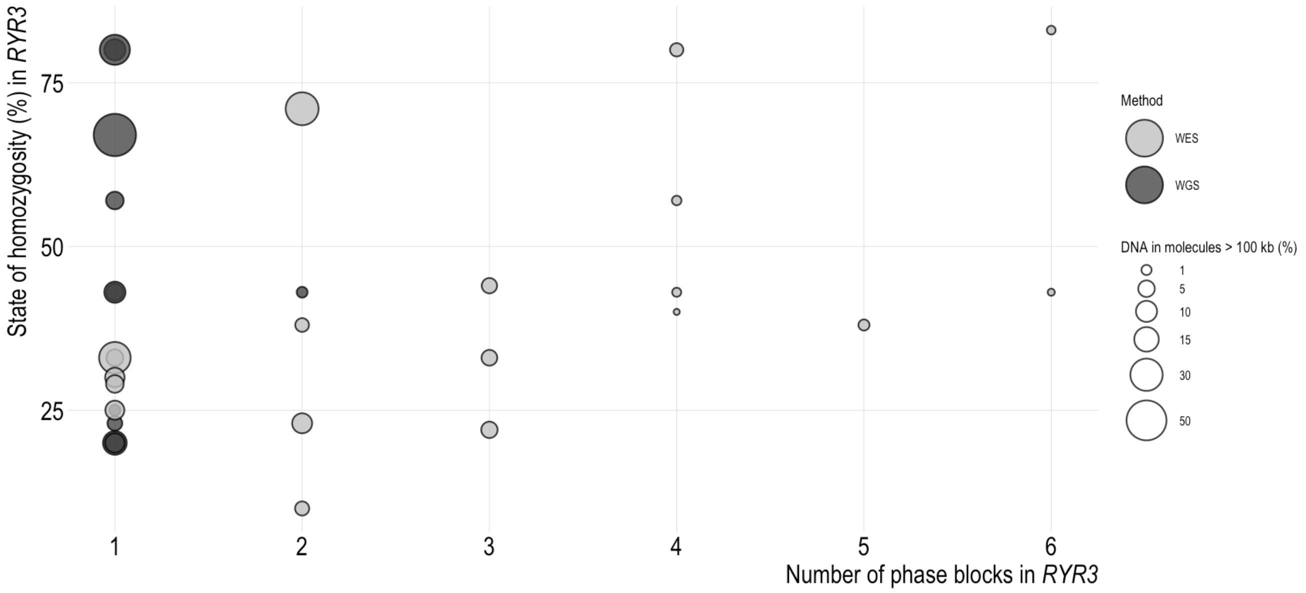
State of homozygosity (%) in RYR3
75
50
25
1
2
3
4
5
6
Number of phase blocks in RYR3
Method
WES
WGS
DNA in molecules > 100 kb (%)
1
5
10
15
30
50

f

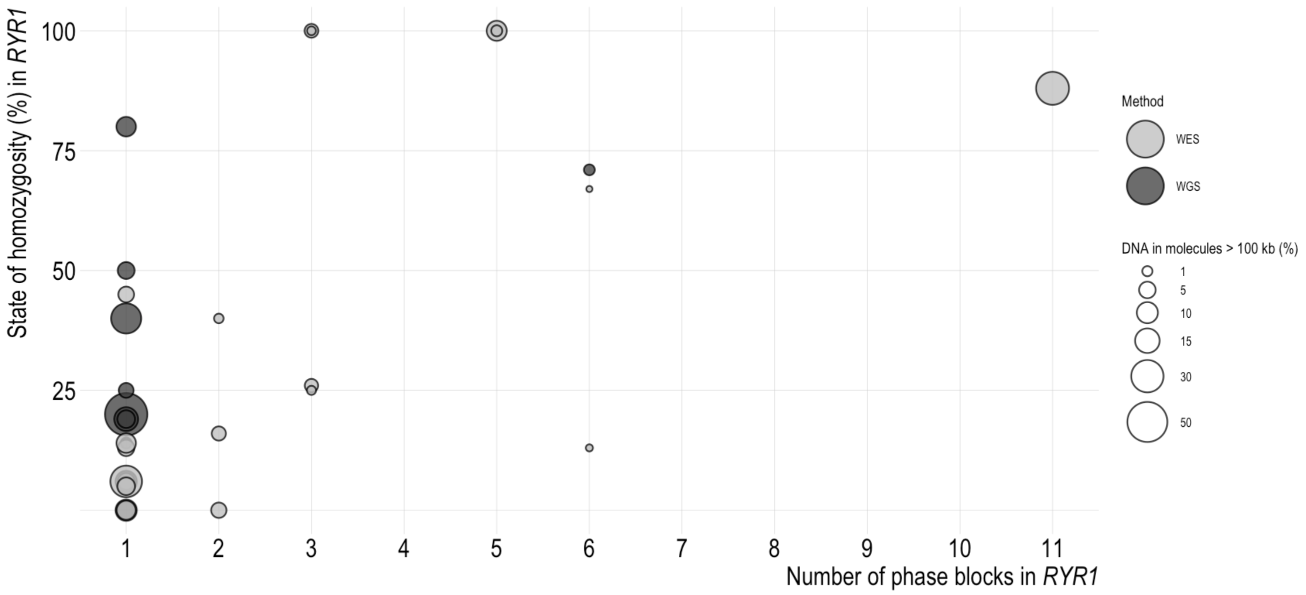
State of homozygosity (%) in RYR1
100
75
50
25
1
2
3
4
5
6
7
8
9
10
11
Number of phase blocks in RYR1
Method
WES
WGS
DNA in molecules > 100 kb (%)
1
5
10
15
30
50

g

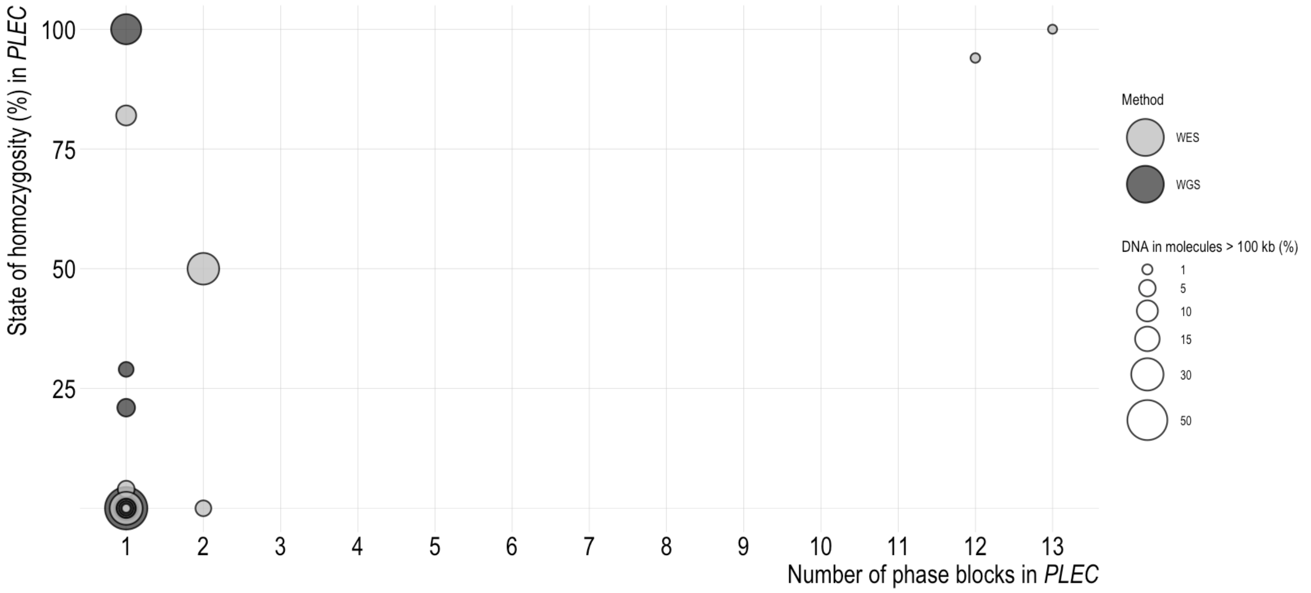

State of homozygosity (%) in *PLEC*
100
75
50
25
1
2
3
4
5
6
7
8
9
10
11
12
13
Number of phase blocks in *PLEC*
Method
WES
WGS
DNA in molecules > 100 kb (%)
1
5
10
15
30
50

h

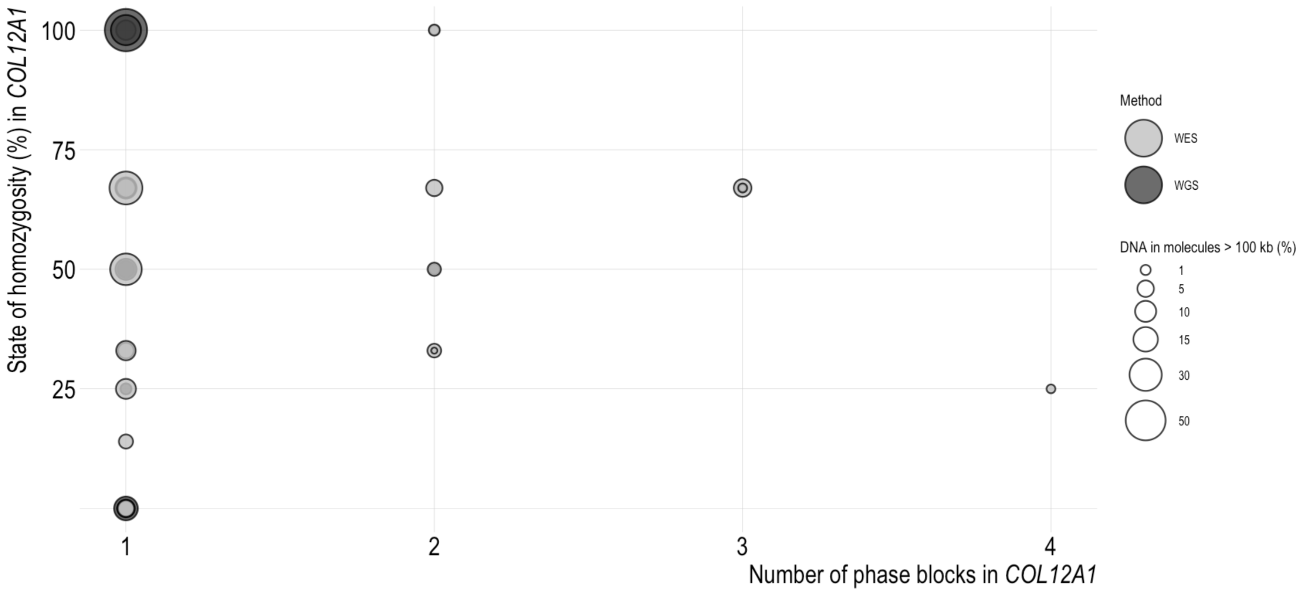

State of homozygosity (%) in *COL12A1*
100
75
50
25
1
2
3
4
Number of phase blocks in *COL12A1*
Method
WES
WGS
DNA in molecules > 100 kb (%)
1
5
10
15
30
50

i

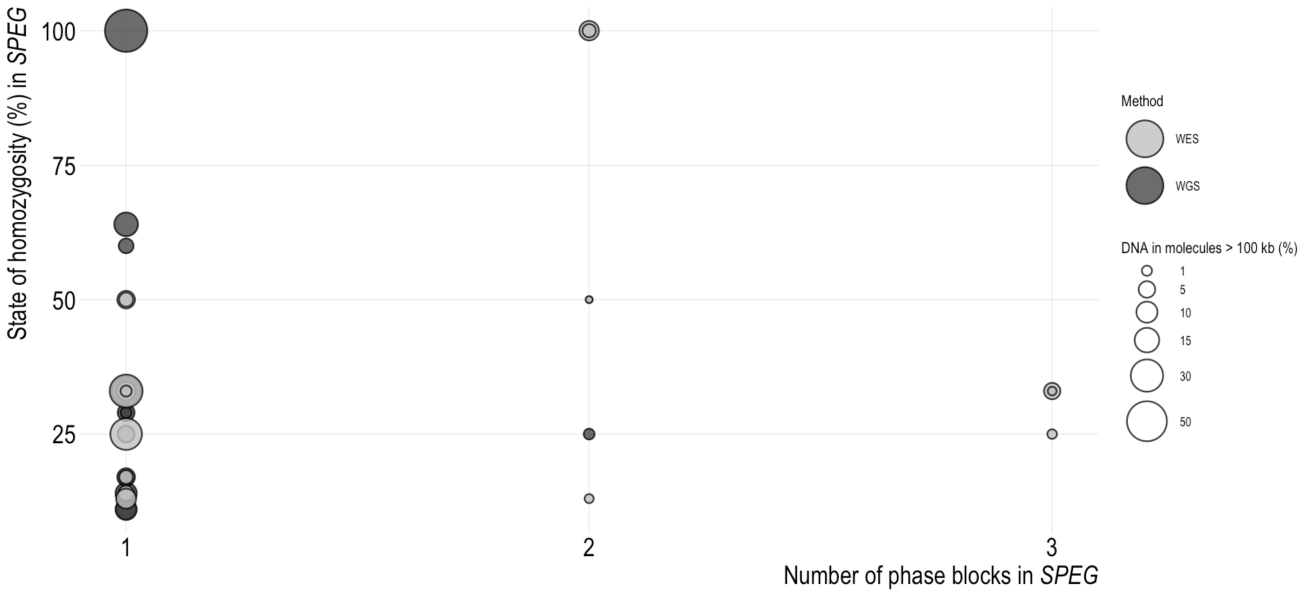

State of homozygosity (%) in *SPEG*
100
75
50
25
1
2
3
Number of phase blocks in *SPEG*
Method
WES
WGS
DNA in molecules > 100 kb (%)
1
5
10
15
30
50

Figure S1. The individual gene plots (a-i) show in how many phase blocks the gene is divided in each sample. The phase block number can be increased because of fragmented DNA or a high degree of homozygosity in the sample. Overall, the whole exome sequenced (WES) samples have more phase blocks than the whole genome sequenced (WGS) samples.

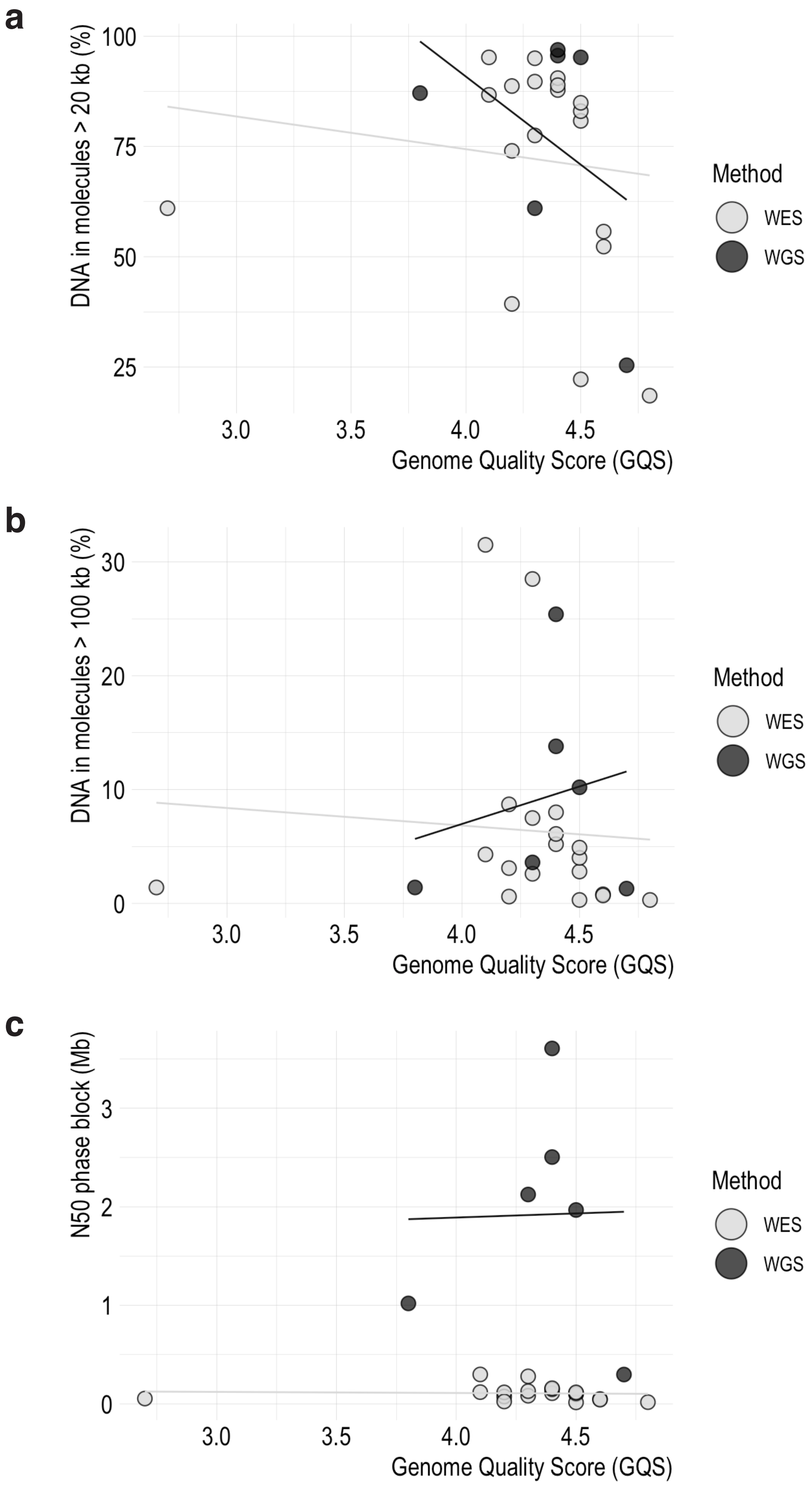

Figure S2. The DNA integrity analysis. The lines represent the mean values for whole exome sequenced (WES) and whole genome sequenced (WGS) samples. There was no correlation between genome quality score (GQS) and the LongRanger algorithm calculated a) percentage of long DNA molecules >20kb, b) percentage of long DNA molecules >100kb, or c) N50 phase blocks. WGS samples performed better and had larger N50 phase block numbers.

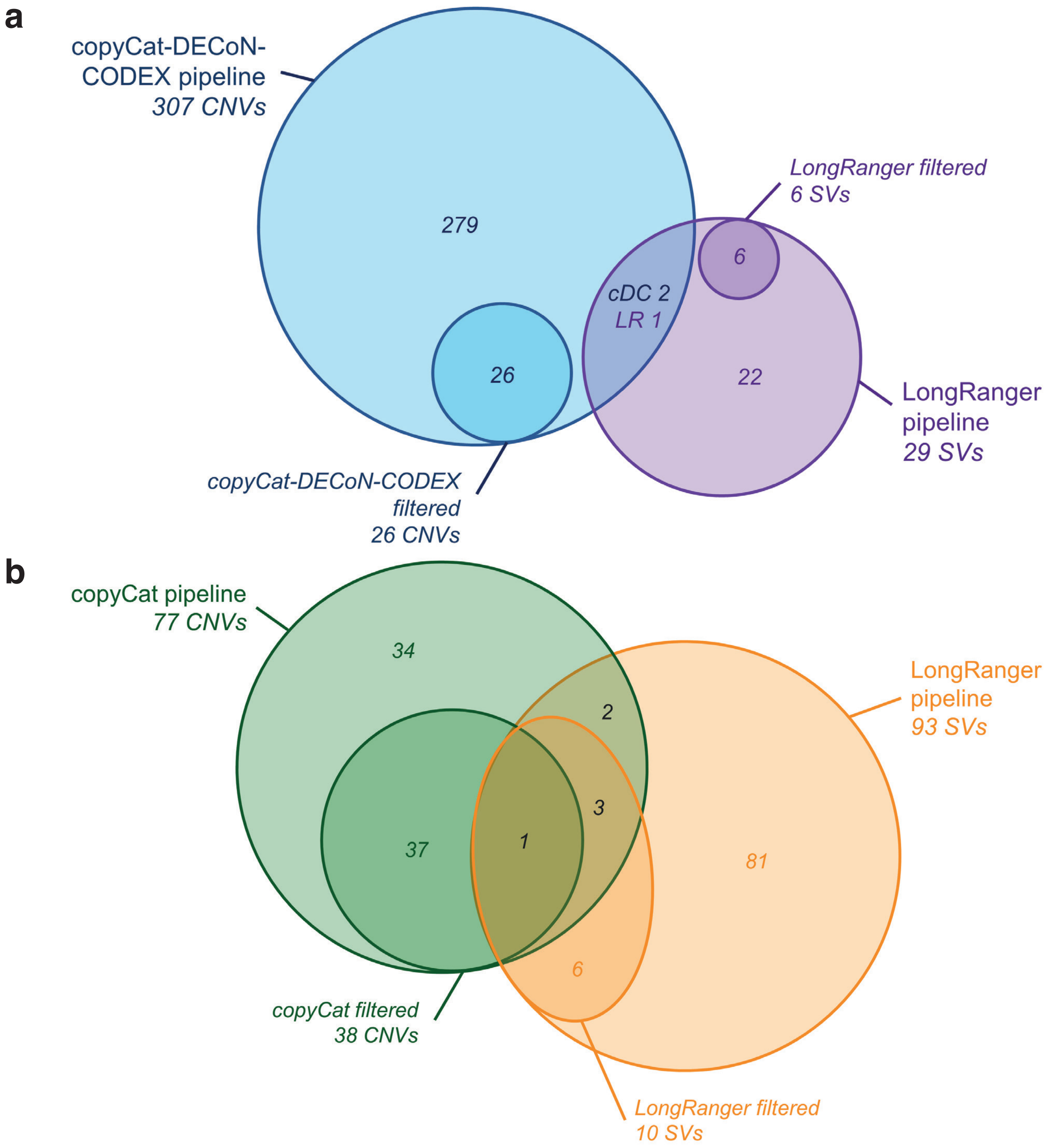

Figure S3. Venn diagrams show the number of called structural variants (SVs) and the number of SVs identified by both short-read-based and linked-read-based methods. a) A short-read-based copyCat-DECoN-CODEX pipeline called 307 copy number variants (CNVs) and the linked-read-based LongRanger pipeline called 29 SVs in the whole exome sequenced (WES) samples in 343 neuromuscular disorder genes. One LongRanger SV was also identified by the copyCat-DECoN-CODEX pipeline (called as two CNVs) when reciprocal ≥ 20% overlap was required. b) The copyCat pipeline identified 77 CNVs and LongRanger 93 SVs in whole genome sequenced (WGS) samples. Six SVs were identified with both methods. For a) and b) numbers of filtered SVs are also shown.

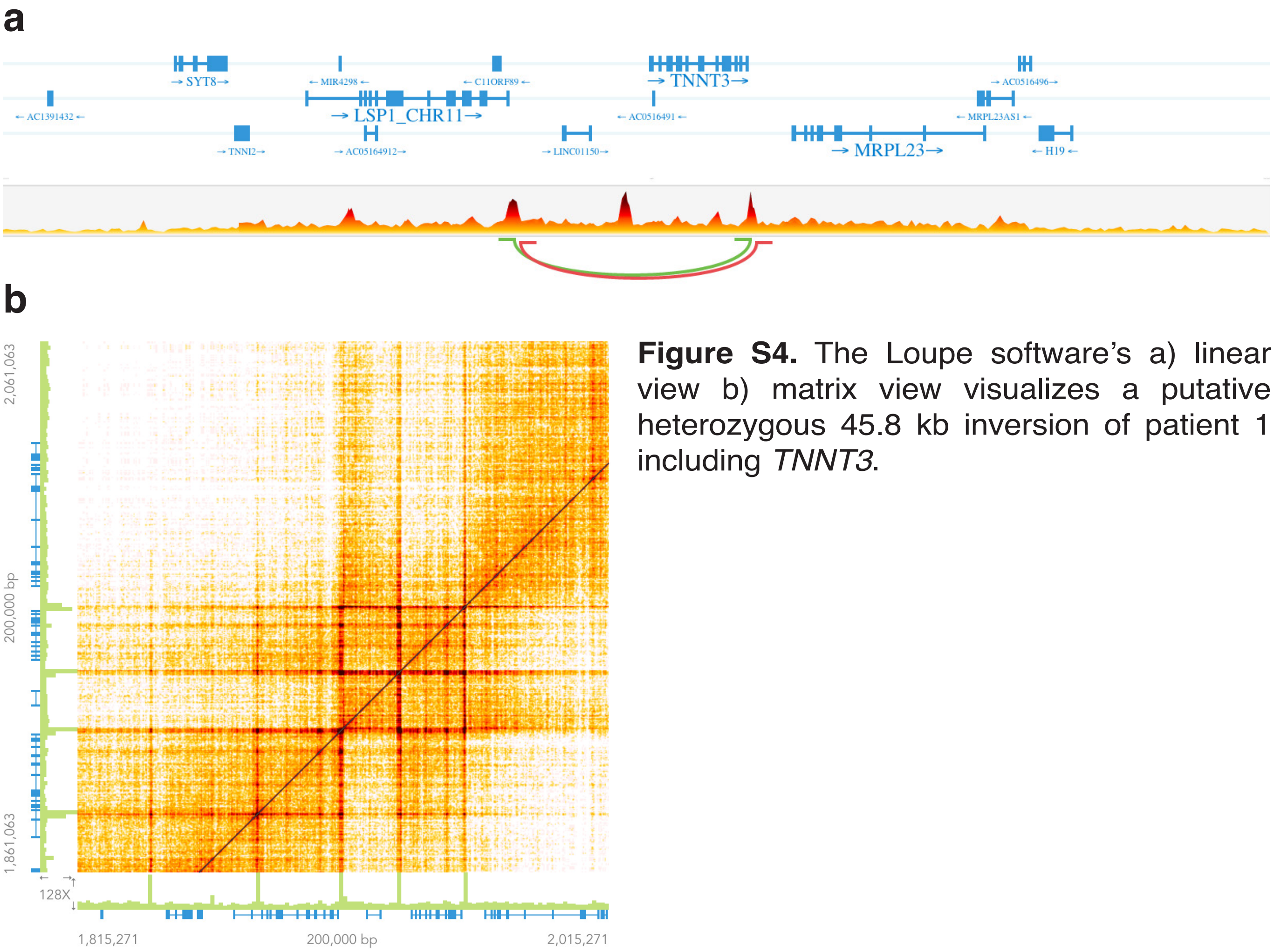

Figure S4. The Loupe software's a) linear view b) matrix view visualizes a putative heterozygous 45.8 kb inversion of patient 1 including *TNNT3*.

Patient descriptions

Patient 1 is comprehensively described in [1].

Patient 2 is a male patient with an unusual combination of congenital myopathy, dystrophic features and nemaline bodies in a muscle biopsy and muscle weakness more pronounced in the lower than in the upper limbs. He has immigrated from another country, where active physiotherapy and other supportive treatments have been lacking. The patient lost ambulation during a period of fever at the age of 5 years and started using a wheelchair. The infection was thought to be polio, but this remained unverified. He has scoliosis and has been using a ventilator since the age of 13 yrs.

Patient 3 is a female patient with congenital global muscle weakness, delayed motor milestones, joint hyperlaxity and cognitive difficulties. An EMG showed mild myopathic features and muscle MRI showed non-specific generalized muscle atrophy or hypotrophy. MRI of the brain gave normal findings. Light and electron microscopy of a muscle biopsy taken at the age of 2 years showed non-specific findings and no nemaline bodies.

A routine aCGH yielded normal findings. Previously done panel sequencing followed by linked-read sequencing revealed the patient to be heterozygous for a *CFL2*:NM_138638:exon2:c.70_72del:p.24_24del. However, analysis of linked-read sequencing data did not reveal any other rare *CFL2* variant, nor a copy number variation that would be compatible with the recessive mode of inheritance. The *CFL2* variant was not identified in the sample of the patient's father, while the DNA sample of the patient's mother was of poor quality and not suitable for Sanger sequencing.

Patient 4 is comprehensively described in [2].

Patient 5 is comprehensively described in [3].

Patient 6 is comprehensively described in [4].

Patient 7 is a male patient with sporadic adult-onset distal myopathy. A previously performed short-read WES was also negative. Relatives are not available for the analysis.

Patient 8 is a male patient with sporadic adult-onset distal myopathy. During his childhood/adolescence, the limited muscle capacity was attributed to asthma. Around 40 years of age, he manifested a voice modification (laryngeal involvement). A few years later, he manifested a hoarse voice (laryngeal paresis), a scapular girdle involvement (abduction 90 degrees), weakness of finger extensors, mild atrophy of thighs, no face and no oculomotor involvement, and no contractures. The creatine kinase (CK) levels were normal. The muscle biopsy showed type 1 predominance and normal examination of dystrophin, sarcoglycan, dysferlin, and caveolin. No variants in genes of lamin and caveolin have been detected. He has a healthy sister and a clinically uncertain son. His son, in his twenties, has a progressive and isolated laryngeal involvement ascertained by an otorhinolaryngologist (unclear). A very limited amount of DNA was available from the patient.

Patient 9 is a female patient with adult-onset proximal myopathy. A reduced level of calpain3 protein was observed by western blotting but *CAPN3* variants have not been identified. A very limited amount of DNA was available from the patient.

Patient 10 is a female patient with late adult-onset distal myopathy. The lower legs are weaker than the hands. Relatives are not available for the analysis.

Patient 11 is a male patient with late adult-onset distal myopathy. The lower legs are weaker than the hands. He has mildly increased CK levels and a rimmed vacuolar pathology +/-. Around 60 years of age, he experienced difficulty in walking. Neurological examination showed severe steppage more accentuated on the right side. No significant muscle weakness in other districts. A few years later, a muscle biopsy from the right tibialis anterior showed variation in fiber size, nuclear centralization, and a few rimmed vacuoles. IIC (dystrophin, dysferlin, laminin, sarcoglycan, and emerin) was normal. Tibialis anterior muscle biopsy was repeated from the left leg, but unfortunately, many technical artifacts were present; however, we observed variations in fiber size and some nuclear internalization. No obvious rimmed

vacuoles seemed to be present. In his sixties, a CT of his lower limbs showed hypotrophy with fibro-adipous substitution of the posterior muscles (right calf) and of the left anterior tibial. CK level ranged from 400 to 800. A very limited amount of DNA was available from the patient.

Patient 12 is a male patient with adult-onset distal myopathy. The lower legs are weaker than the hands. Relatives are not available for the analysis.

Patient 13 is a male patient with sporadic late adult-onset distal myopathy. The lower legs are weaker than the hands. Previously done panel sequencing followed by linked-read sequencing revealed the patient to be heterozygous for a *POLG*:NM_002693:exon13:c.G2243C:p.W748S. Variants in *POLG* are causative for mitochondrial recessive ataxia syndrome (MIRAS). However, no other *POLG* variants have been identified in this patient.

References

1. Pelin, K. *et al.* Novel Compound Heterozygous Splice-Site Variants in TPM3 Revealed by RNA Sequencing in a Patient with an Unusual Form of Nemaline Myopathy: A Case Report. *J. Neuromuscul. Dis.* **10**, 1–8 (2023).
2. Savarese, M. *et al.* Out-of-Frame Mutations in ACTN2 Last Exon Cause a Dominant Distal Myopathy With Facial Weakness . *Neurol. Genet.* **7**, e619 (2021).
3. Välipakka, S. *et al.* Copy number variation analysis increases the diagnostic yield in muscle diseases. *Neurol. Genet.* **3**, e204 (2017).
4. Hackman, P. *et al.* Dominant Distal Myopathy 3 (MPD3) Caused by a Deletion in the HNRNPA1 Gene. *Neurol. Genet.* **7**, 1–10 (2021).
